# Reduction of biologic pricing following biosimilar introduction: Analysis across 57 countries and regions, 2012–19

**DOI:** 10.1371/journal.pone.0304851

**Published:** 2024-06-06

**Authors:** Hui-Han Chen, Tatenda Yemeke, Sachiko Ozawa

**Affiliations:** 1 Division of Practice Advancement and Clinical Education, UNC Eshelman School of Pharmacy, University of North Carolina, Chapel Hill, NC, United States of America; 2 Department of Maternal and Child Health, UNC Gillings School of Global Public Health, University of North Carolina, Chapel Hill, NC, United States of America; Kings College Hospital, UNITED KINGDOM

## Abstract

**Objective:**

To evaluate the impact of the entry of biosimilars on the pricing of eight biologic products in 57 countries and regions.

**Methods:**

We utilized an interrupted time series design and IQVIA MIDAS® data to analyze the annual sales data of eight biologic products (adalimumab, bevacizumab, epoetin, etanercept, filgrastim, infliximab, pegfilgrastim, and trastuzumab) across 57 countries and regions from January 1, 2012, to December 31, 2019. We examined the immediate and long-term changes in biologics ex-manufacturer pricing following the entry of biosimilars to the market.

**Results:**

Following the entry of biosimilars, the average price per dose of biologic product was immediately reduced by $438 for trastuzumab, $112 for infliximab, and $110 for bevacizumab. The persistent effect of biosimilars’ market entry led to further reductions in price per dose every year: by $49 for adalimumab, $290 for filgrastim, $21 for infliximab, and $189 for trastuzumab. Similarly, we analyzed the impact of biosimilars on four biologics’ prices in the US, where the prices of three biologics significantly decreased every year, with filgrastim, pegfilgrastim, and infliximab decreasing by $955, $753, and $104, respectively.

**Conclusions:**

The introduction of biosimilars has significantly reduced the prices of biologics both globally and in the US. These findings not only demonstrate the economic benefits of increasing biosimilar utilization, but also emphasize the importance of biosimilars in controlling healthcare costs. Policies should aim to expand the availability of biosimilars to counteract the exponential growth of medical spending caused by the use of biologics.

## Introduction

Biologics are pharmaceutical products that contain polysaccharides, proteins, nucleic acids, or a mixture of these components [1, 2]. These medications can be produced through modern biotechnology in genetically engineered cells, which differs from conventional medications in many aspects [3, 4]. For example, the active component of biologics is usually a part of a large macromolecule, whereas small-molecule medicines often have a unique entity as active pharmaceutical ingredients [2]. The combination of various substances can make the structure of the medications challenging to characterize, as proteins and polypeptides have different numbers of identical parts in their structures and the surface polysaccharides can affect the folding patterns of the large macromolecule [2, 5–7]. Since macromolecules are heterogeneous, the impurity profile of biologics depends on the manufacturing processes and can vary across different batches, which requires more quality tests with higher manufacturing costs than small-molecule medications [2, 8].

Biosimilars are a group of biologics highly similar to their reference products in structure, treatment efficacy, safety profile, product purity, chemical identity, and bioactivity [9, 10]. However, unlike generic medicines in conventional small-molecule drugs, where generic medicines have to be chemically equivalent to the brand medicine, biosimilars can differ from their reference products regarding some inactive substances, such as stabilizers or buffers [11]. These minor differences should not result in clinically meaningful discrepancies between biosimilars and their reference products [12, 13]. While many biologics exist across various therapeutic areas, only a small fraction have biosimilar products that are commercially available. As of December 2022, 39 biosimilars have been approved by the US Food and Drug Administration (FDA), and less than half of these approved biosimilars are commercially available [14]. These biosimilars approved by the FDA corresponded to 11 products, including bevacizumab, etanercept, epoetin alfa, trastuzumab, adalimumab, insulin glargine, ranibizumab, pegfilgrastim, filgrastim, infliximab, and rituximab.

The growth of medication expenditures in the US between 2013 and 2017 was largely driven by biological products [8]. In fact, while less than 2% of prescriptions filled in the US in 2017 were categorized as biologics, they represented almost 40% of all medication expenditures in that year [15]. While the utilization of biologics has exponentially increased over the past decade, their growing costs have limited patients’ access to these highly effective treatment options. For instance, research suggests that in the US, the average total medical expenditure per person for biologic-containing disease-modifying antirheumatic drugs (DMARDs) was approximately $26,217, with out-of-pocket expenses averaging $1,484 [16]. In contrast, conventional DMARDs only incurred an average total medical expenditure of $5,389, with out-of-pocket expenses averaging $396 per person [16]. Prior studies also show that insurance restrictions, including the need for prior authorization and higher out-of-pocket costs, have hindered the use of biologics [17–20]. To improve patient access to effective biologic treatments, it is imperative to contain the rising healthcare expenditures associated with broader utilization of biologics, which are generally considered more expensive than traditional small molecule medicines [8, 21]. The development and use of biosimilars come with great hope for price reductions and improved access to effective treatment options [22, 23].

Despite the growing body of literature on the impact of biosimilars on medication prices, there is currently limited evidence on the change of prices at the manufacturer and drug distribution channel level (e.g., hospitals and retail pharmacies) [16, 24–26]. Most prior studies examined the prices of biologics in high-income countries and only focused on a few select products [16, 24–26]. The estimated effects of biosimilars on medication prices in low- and middle-income countries (LMICs) and whether market entry of approved biosimilars is significantly correlated with price changes for the existing reference products remain unknown. Additionally, as most of the previous research focused on out-of-pocket costs paid by patients using individual pharmacy and outpatient insurance claims, changes in the marketplace activity between manufacturers and drug distribution channels remain unclear [27, 28]. Although a few studies suggest price reductions for biologics following market entry of biosimilars, these have been primarily based on public or insurance claims [23, 27, 29, 30]. The underlying weakness of claims data not showing the negotiations and discounts between healthcare providers and insurers may limit the ability of these studies to reflect the actual transaction behaviors due to the entry of biosimilars.

We estimated the associations between biosimilar availability across 57 countries and regions and the global marketplace transaction prices between the drug manufacturers and the distribution channels. The purposes of this study were to examine (1) the influence of biosimilars on ex-manufacturer drug prices around the world and (2) the short-term and long-term effects on the prices of biologics due to the market entry of biosimilars.

## Methods

To understand the impact of introducing biosimilars on global biologic prices, we conducted a retrospective interrupted time series (ITS) analysis assessing yearly prices of biologics from January 2012 to December 2019. We utilized the global sales data of biologics in the IQVIA MIDAS® data, which were obtained under license from IQVIA and reflect estimates of marketplace activity. The data used in this analysis was retrieved on February 5, 2020, for the time period between January 1, 2012 and December 31, 2019. IQVIA national audits and MIDAS reflect local industry standard sources of pack prices, which might be list price or average invoice price, depending upon the country and the available information; they do not reflect net prices realized by the manufacturers or achieved by the payer. Most of the drugs in question are purchased by hospitals, which can receive commercial rebates, regulated rebates, and claw-backs, details of which are normally kept confidential. Sales values reflected in these IQVIA audits are calculated by applying such relevant pricing to the product volume data collected for and reflected in such audits. In addition, to allow the national audit sales values to be viewed at a common sales level, MIDAS applies a single average industry margin to the locally reported values. The drug price provided is an estimated price, and its intended function is to convert volumes to sales–this estimated price is not intended to be used as a metric in its own right. Understanding market dynamics in each country is required to make cross-country pricing comparisons. Prices are collected from different sources in each country; as such, completeness can vary, although likely not to affect the overall conclusions. The prices used (ex-manufacturing, list price) are normalized values using the same definition between countries. The MIDAS data has been utilized in numerous global studies, providing valuable insights into its reliability and validity in analyzing medication prices [31–35].

The MIDAS database contained annual sales data of medicines collected across different distribution channels (e.g., wholesaler, retail, hospital, and drugstore) across 75 countries and regions (Central America region includes Guatemala, Honduras, El Salvador, Nicaragua, Costa Rica, and Panama; French West Africa region comprises Benin, Burkina Faso, Cameroon, Chad, Gabon, Guinea, Ivory Coast, Mali, Niger, Republic of the Congo, Senegal, and Togo). We applied the 2022 World Bank country-income classifications to the countries in the IQVIA MIDAS database [36]. In addition, we applied World Health Organization (WHO) regional grouping classifications and noted whether countries were members of the Organisation for Economic Co-operation and Development (OECD) [37, 38].

Prices of each of eight biologics (adalimumab, bevacizumab, epoetin, etanercept, filgrastim, infliximab, pegfilgrastim, and trastuzumab) were extracted across 57 countries and regions where the biologics were available on the market. The countries and regions included in the analysis were the following: Argentina, Australia, Austria, Belarus, Belgium, Bosnia, Brazil, Bulgaria, Canada, Chile, Colombia, Croatia, Egypt, Estonia, Finland, France, Germany, Greece, Hungary, India, Indonesia, Ireland, Italy, Japan, Kazakhstan, Korea, Latvia, Lebanon, Lithuania, Luxembourg, Malaysia, Mexico, Morocco, Netherlands, Norway, Pakistan, Peru, Philippines, Poland, Portugal, Romania, Russia, Serbia, Singapore, Slovakia, Slovenia, Spain, Sweden, Switzerland, Taiwan, Thailand, Tunisia, Turkey, United Arab Emirates, United Kingdom, United States of America, and Vietnam. To better understand the biologic pricing within each of the countries and regions, we analyzed the average medicine price of each of the eight products across all distribution channels (e.g., hospitals and retail pharmacies) in the country. As each of the eight biologic products was available in different dosage formulations, we standardized the dose of the biologics to its most commonly used dosage for ease of comparison. First, we identified the standardized dose of each biologic based on the biologic’s dosage formulation that appeared in the data source most frequently (i.e., 40 mg for adalimumab, 100 mg for bevacizumab, 10,000 IU for epoetin, 25 mg for etanercept, 300 mcg for filgrastim, 100 mg for infliximab, 6 mg for pegfilgrastim, and 150 mg for trastuzumab), and calculated the relative proportion of each dosage formulation to the biologic’s standardized dose. Then, we standardized the medicine price to the most used dosage of each of the eight biologics by multiplying the relative proportion derived above by the medicine’s original price. A similar approach for controlling standardized doses in analyzing changes in medication prices has been employed and validated in a prior study [27].

Our study began with descriptive statistics to examine the distribution of biologics characteristics in countries and regions where both reference and biosimilar biologics were available on the market. To understand the impact of introducing biosimilars on the ex-manufacturer price of biologics, we excluded countries and regions that introduced biosimilars before 2012, as our MIDAS data spanned the period from 2012 to 2019. Within each country or region, the year of biosimilar’s market entry for each of the eight biologics was assessed based on the first year the biosimilar became commercially available in the MIDAS data. We then employed a quasi-experimental design using ITS analysis with an ordinary least squares regression to examine the influence of the market entry of biosimilars on the annual average biologic price across all countries and regions where the biosimilar was available on the market. The ITS analysis assessed both the immediate effects and long-term impact of biosimilar entry on prices by examining changes in the trend (slope) of average biologic prices. The ITS analysis incorporated time as a continuous variable indicating the number of years since the biosimilar’s market entry, interruption as a binary variable indicating whether a biosimilar is available for the reference biologic, and an interaction term between time and interruption. Additionally, the ITS analysis also controlled for the number of standardized doses sold for each biologic. A mixed-effects model with a random intercept and slopes for the regression was performed to account for the autocorrelation of biologic price within each country and region. Separate ITS analyses were performed to examine the variation in the magnitude of price change by country income levels. We also evaluated the influence of the introduction of biosimilars on the prices of their reference products.

In addition to the global analysis, we separately examined the impact of biosimilars in the United States of America (US). We assessed the medicine prices of four biologics (epoetin, filgrastim, infliximab, and pegfilgrastim), where the biosimilar forms of each of the four biologics were commercially available in the US. A similar mixed-effects ITS analysis was applied to evaluate the impact of introducing biosimilars on average biologic price. All statistical analyses were performed using SAS version 9.4 (SAS Institute Inc, Cary, NC).

## Results

Among the 75 countries and regions in the MIDAS database, eight biologics were commercially available in 64 countries, and at least one biosimilar of the eight biologics was available in 57 countries. Infliximab had the most widespread availability in biosimilar forms compared to the other seven biologics, where biosimilar infliximab was available in 43 countries and regions with a median market entry date in 2015 (Table 1). Biosimilar trastuzumab was available in 31 countries and regions, and biosimilar pegfilgrastim was available in 27 countries. Bevacizumab had the lowest number of countries and regions where biosimilar was available—only seven countries had biosimilar bevacizumab in 2019. Most biosimilars were commercially available (i.e., approved and used in the market) in either high-income or upper middle-income countries and were not commonly available in low-income countries. Member of OECD countries that had biosimilars in their markets varied widely across biosimilars. More than 80% of all available biosimilar adalimumab, etanercept, and pegfilgrastim were available in OECD countries. In contrast, filgrastim had the lowest proportion of availability among OECD countries, with 57.9% availability in countries that were not OECD members. The commercial availability of approved biosimilars differed across WHO regions. Countries in the European Region had a large biosimilars market, where 92.4%, 55.6%, 80.0%, 62.8%, 81.5%, and 74.2% of biosimilar adalimumab, epoetin, etanercept, infliximab, pegfilgrastim, and trastuzumab were available in the European Region, respectively.

**Table 1:** Characteristics of commercially available biosimilars across 57 countries and regions.

Biosimilar	Pharmacologic Category	Number of Countries	Median Year of Introduction (IQR)	Country Income	OECD Countries	WHO Region
**Adalimumab**	Antirheumatic, Disease Modifying; Gastrointestinal Agent; Monoclonal Antibody; Tumor Necrosis Factor (TNF) Blocking Agent	26	2018 (2018–2018)	High income: 23 (88.5%) Upper-middle income: 3 (11.5%)	80.8%	EMR: 2 (7.7%) EUR: 24 (92.3%)
**Bevacizumab**	Monoclonal Antibody; Antineoplastic Agent, Vascular Endothelial Growth Factor (VEGF) Inhibitor	7	2019 (2019–2019)	High income: 4 (57.1%) Upper-middle income: 2 (28.6%) Lower-middle income: 1 (14.3%)	57.1%	AMR: 2 (28.6%) EUR: 1 (14.3%) SEAR: 2 (28.6%) WPR: 2 (28.6%)
**Epoetin**	Colony Stimulating Factor; Erythropoiesis-Stimulating Agent (ESA); Hematopoietic Agent	9	2018 (2014–2018)	High income: 4 (44.4%) Upper-middle income: 4 (44.4%) Lower-middle income: 1 (11.1%)	44.4%	AMR: 3 (33.3%) EUR: 5 (55.6%) WPR: 1 (11.1%)
**Etanercept**	Antirheumatic, Disease Modifying; Tumor Necrosis Factor (TNF) Blocking Agent	25	2016 (2016–2017)	High income: 24 (96.0%) Lower-middle income: 1 (4.0%)	88.0%	AMR: 1 (4.0%) EUR: 20 (80.0%) SEAR: 1 (4.0%) WPR: 3 (12.0%)
**Filgrastim**	Colony Stimulating Factor; Hematopoietic Agent	19	2015 (2013–2015)	High income: 7 (36.8%) Upper-middle income: 8 (42.1%) Lower-middle income: 4 (21.1%)	42.1%	AMR: 5 (26.3%) EMR: 4 (21.1%) EUR: 4 (21.1%) SEAR: 1 (5.3%) WPR: 5 (26.3%)
**Infliximab**	Antirheumatic, Disease Modifying; Gastrointestinal Agent; Immunosuppressant Agent; Monoclonal Antibody; Tumor Necrosis Factor (TNF) Blocking Agent	43	2015 (2015–2015)	High income: 28 (65.1%) Upper-middle income: 11 (25.6%) Lower-middle income: 4 (9.3%)	60.5%	AMR: 5 (11.6%) EMR: 4 (9.3%) EUR: 27 (62.8%) SEAR: 1 (2.3%) WPR: 6 (14.0%)
**Pegfilgrastim**	Colony Stimulating Factor; Hematopoietic Agent	27	2018 (2018–2019)	High income: 24 (88.9%) Upper-middle income: 2 (7.4%) Lower-middle income: 1 (3.7%)	81.5%	AMR: 3 (11.1%) EUR: 22 (81.5%) WPR: 2 (7.4%)
**Trastuzumab**	Monoclonal Antibody; Antineoplastic Agent, Anti-HER2;	31	2018 (2018–2019)	High income: 26 (83.9%) Upper-middle income: 4 (12.9%) Lower-middle income: 1 (3.2%)	77.4%	AMR: 2 (6.5%) EMR: 1 (3.2%) EUR: 23 (74.2%) SEAR: 2 (6.5%) WPR: 3 (9.7%)

AFR: African Region; AMR: Region of the Americas; SEAR: South-East Asian Region; EUR: European Region; EMR: Eastern Mediterranean Region; WPR: Western Pacific Region. Source: This is based on internal analysis by Hui-Han Chen, Tatenda Yemeke, and Sachiko Ozawa using data from the following source: IQVIA MIDAS Annual Sales for the period 2012–2019 reflecting estimates of real-world activity. Copyright IQVIA. All rights reserved.

This study identified 16,691 marketplace activity records across the 57 countries and regions where any of the eight biosimilars were available between January 2012 and December 2019. Among these, 2,167 were related to adalimumab, 326 were related to bevacizumab, 3,197 were related to epoetin, 3,112 were related to etanercept, 2,553 were related to filgrastim, 2,432 were related to infliximab, 1,225 were related to pegfilgrastim, and 1,679 were related to trastuzumab. We observed an increase in consumption volume, measured by the total doses of both reference and biosimilar products sold each year, across adalimumab, bevacizumab, epoetin, etanercept, infliximab, and pegfilgrastim. Compared to the year when biosimilars became commercially available, the total doses sold after one year of biosimilar introduction increased by 16.3% for adalimumab, 29.7% for bevacizumab, 10.8% for epoetin, 7.3% for etanercept, 6.8% for infliximab, and 7.0% for pegfilgrastim. In contrast, the consumption of filgrastim and trastuzumab decreased by 7.2% and 3.4%, respectively, one year after biosimilar introduction. In addition, we have observed a reduction in demand for reference products across seven biologics: a decrease of 19.8% for adalimumab, 9.6% for epoetin, 14.8% for etanercept, 20.0% for filgrastim, 1.3% for infliximab, 13.8% for pegfilgrastim, and 17.8% for trastuzumab. The only exception is bevacizumab, where demand for the reference product increased by 6.5% following the introduction of biosimilars.

For each biologic, the proportion sold that was biosimilars was 5.7% for adalimumab, 0.4% for bevacizumab, 3.0% for epoetin, 15.9% for etanercept, 15.1% for filgrastim, 13.5% for infliximab, 3.2% for pegfilgrastim, and 2.3% for trastuzumab. The proportion of biologics sold after the market entry of biosimilars, measured by the number of doses sold after biosimilars became available divided by the total number of doses sold before and after the market entry of biosimilars for each biologic, varied widely across the eight biologics: with 32.9% for adalimumab, 16.2% for bevacizumab, 32.2% for epoetin, 47.1% for etanercept, 59.8% for filgrastim, 63.7% for infliximab, 23.7% for pegfilgrastim, and 28.3% for trastuzumab. Overall, the utilization of biosimilars for seven biologics (adalimumab, epoetin, etanercept, filgrastim, infliximab, pegfilgrastim, and trastuzumab) has increased over time. Up until the most recent year of the data (2019), the proportion of biosimilar sold among each biologic has reached 54.4%, 19.5%, 60.0%, 67.7%, 70.6%, 66.7%, and 55.6% for adalimumab, epoetin, etanercept, filgrastim, infliximab, pegfilgrastim, and trastuzumab, respectively. The average prices of biologics per unit of standardized dose sold before the introduction of biosimilars were $273.2, $266.7, $733.8, $36.3, $25.2, $347.1, $867.8, and $515.8 for adalimumab, bevacizumab, epoetin, etanercept, filgrastim, infliximab, pegfilgrastim, and trastuzumab, respectively. The average prices of biologics weighted by the quantity of active pharmaceutical ingredients sold after the introduction of biosimilars were $176.7, $235.7, $260.5, $25.4, $21.9, $324.0, $1,026.1, and $457.7 for adalimumab, bevacizumab, epoetin, etanercept, filgrastim, infliximab, pegfilgrastim, and trastuzumab, respectively.

**Fig 1:**
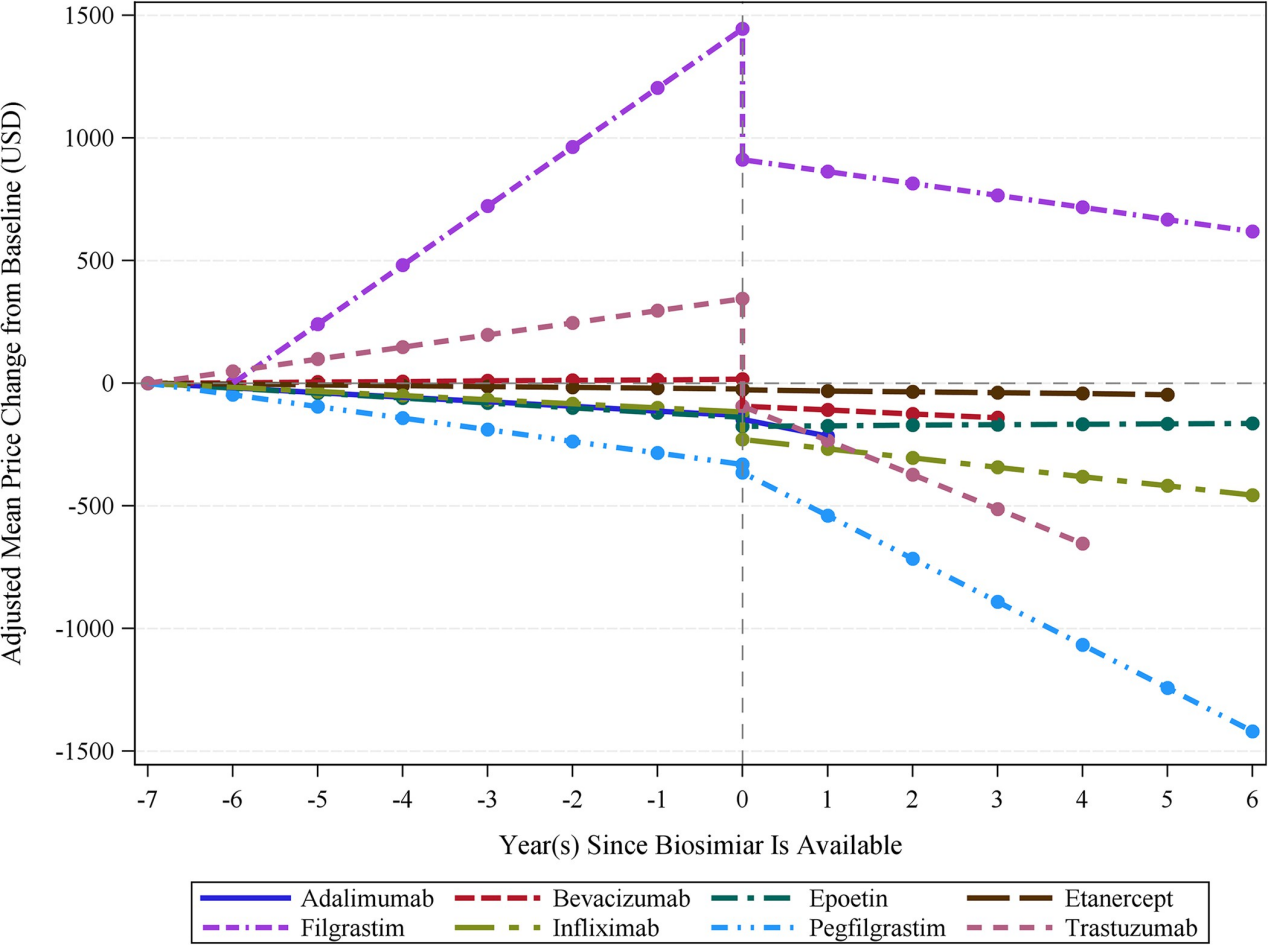
Interrupted time series analysis of biologic prices before and after biosimilar introductions across countries. Source: This is based on internal analysis by Hui-Han Chen, Tatenda Yemeke, and Sachiko Ozawa using data from the following source: IQVIA MIDAS Annual Sales for the period 2012–2019 reflecting estimates of real-world activity. Copyright IQVIA. All rights reserved.

**Fig 2:**
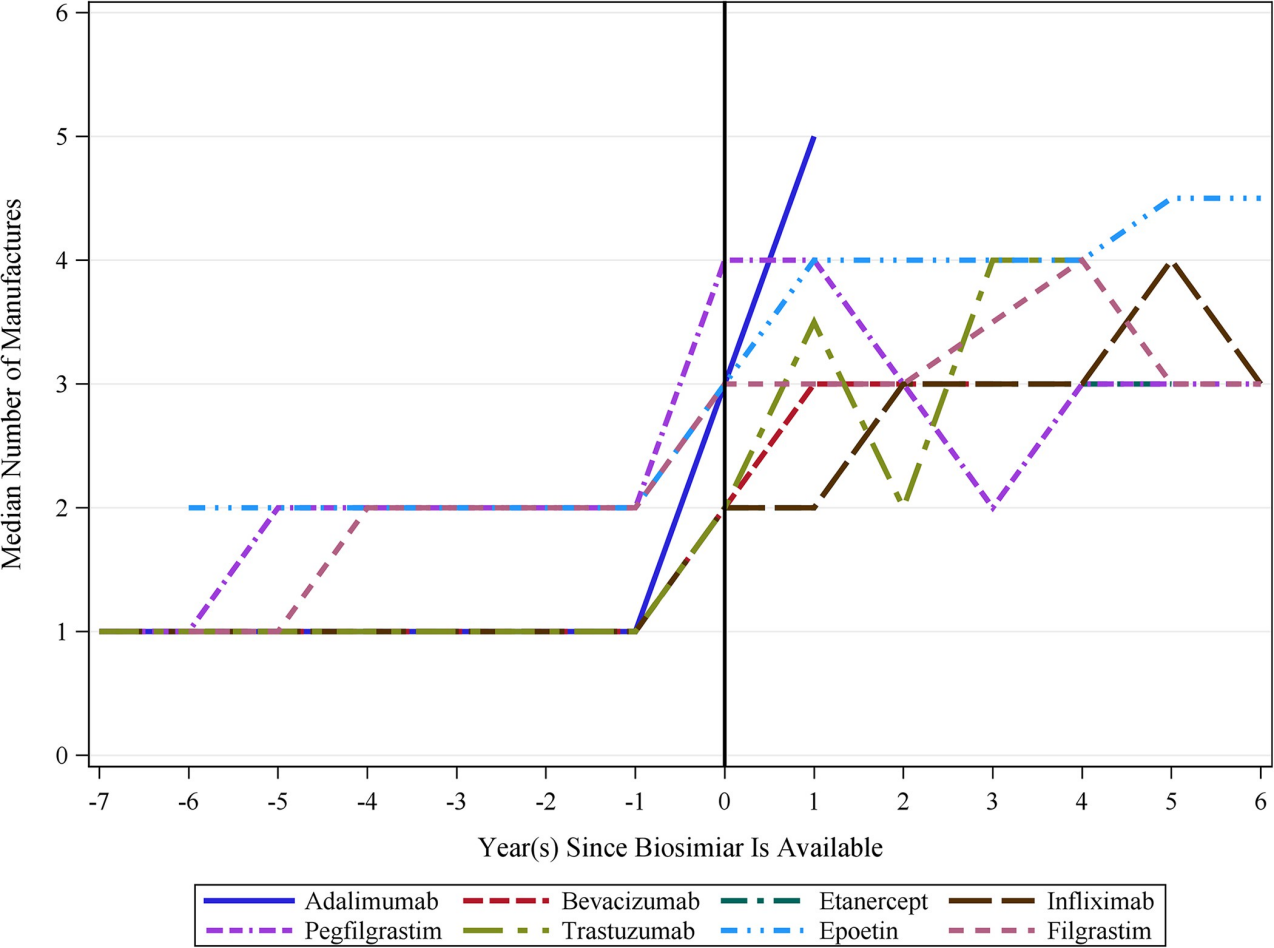
Median number of manufacturers before and after biosimilar introductions across countries. Source: This is based on internal analysis by Hui-Han Chen, Tatenda Yemeke, and Sachiko Ozawa using data from the following source: IQVIA MIDAS Annual Sales for the period 2012–2019 reflecting estimates of real-world activity. Copyright IQVIA. All rights reserved.

The entry of biosimilars in the global biologics market was associated with an immediate reduction in the average price for most of the eight biologics, including reference biologics and biosimilars, examined in this study (Table 2 and Fig 1). The average prices for bevacizumab, infliximab, and trastuzumab were statistically significantly reduced by $110.5 (24.7%), $112.2 (18.4%), and $438.4 (27.7%) per standardized dose in the same year of the market entry of biosimilars. On the other hand, the average prices for adalimumab, epoetin, etanercept, filgrastim, and pegfilgrastim were reduced by $14.8 (5.4%), $35.7 (64.9%), $4.7 (8.7%), $532.8 (63.6%), and $32.5 (3.3%), respectively, but these reductions were not statistically significant. Meanwhile, the data showed that the entry of a biosimilar was associated with a decreasing slope of the price of biologics over time for most of the eight biologics. Compared to the slopes of price changes over time for biologics before the market entry of biosimilars, adalimumab, filgrastim, infliximab, and trastuzumab experienced statistically significant yearly decreases in average biologics price by $49.2 (260.9%), $289.7 (120.3%), $21.2 (127.2%), and $189.3 (484.2%). These reductions in the slopes of price changes, each exceeding 100%, suggest that these biologics initially had a positive slope before the introduction of biosimilars, indicating that their prices were increasing over time. Following the introduction of biosimilars, the slope became negative, suggesting a decrease in price over time. Similarly, bevacizumab, etanercept, and pegfilgrastim also showed a decreasing trend in average biologics prices by $17.7 (849.8%), $0.5 (16.4%), and $128.5 (271.8%), although these reductions were not statistically significant. Furthermore, we observed that the median number of manufacturers of all eight biologics has increased after introducing biosimilars into the market (Fig 2).

**Table 2:** Dose-adjusted results of interrupted time series analyses. All analyses were adjusted for the number of doses sold in a specific year using the following regression model: Price = β_0_+β_1_Time+β_2_Interruption+β_3_(Time x Interruption)+β_4_Doses.

Biologic	Variable	Point Estimate (USD)	Standard Error	P-value
**Adalimumab**	Intercept	272.58	25.15	<0.001
	Time	-18.85	3.73	<0.001
	Interruption	-14.76	14.50	0.3181
	Interaction	-49.18	13.65	<0.01
**Bevacizumab**	Intercept	447.76	56.04	<0.001
	Time	2.36	7.48	0.7614
	Interruption	-110.52	28.89	<0.01
	Interaction	-17.73	26.06	0.5023
**Epoetin**	Intercept	55.09	72.13	0.4479
	Time	-19.91	18.91	0.309
	Interruption	-35.74	72.96	0.6243
	Interaction	21.93	28.61	0.4505
**Etanercept**	Intercept	53.73	6.78	<0.001
	Time	-3.23	0.84	<0.001
	Interruption	-4.68	2.51	0.0807
	Interaction	-0.53	1.00	0.598
**Filgrastim**	Intercept	838.45	305.62	<0.01
	Time	240.82	125.26	0.0546
	Interruption	-532.83	303.54	0.0793
	Interaction	-289.67	130.55	0.0266
**Infliximab**	Intercept	610.98	29.02	<0.001
	Time	-16.68	8.83	0.0594
	Interruption	-112.17	24.45	<0.001
	Interaction	-21.21	8.97	0.0182
**Pegfilgrastim**	Intercept	989.69	154.46	<0.001
	Time	-47.30	14.68	<0.01
	Interruption	-32.54	44.89	0.4686
	Interaction	-128.54	75.08	0.1086
**Trastuzumab**	Intercept	1580.89	104.32	<0.001
	Time	49.28	19.65	0.0143
	Interruption	-438.37	84.83	<0.001
	Interaction	-189.33	68.14	<0.01

Source: This is based on internal analysis by Hui-Han Chen, Tatenda Yemeke, and Sachiko Ozawa using data from the following source: IQVIA MIDAS Annual Sales for the period 2012–2019 reflecting estimates of real-world activity. Copyright IQVIA. All rights reserved.

This study also showed associated reductions in price among the reference biologic products after the market entry of their biosimilars. The average prices of reference biologics were instantly reduced by $63.3 (14.4%), $22.9 (53.9%), $2.0 (3.7%), $359.0 (46.9%), $54.7 (8.9%), and $179.6 (11.3%) for bevacizumab, epoetin, etanercept, filgrastim, infliximab, and trastuzumab, respectively, the same year after their biosimilars entered the market. Similar associations were observed in the long-term trend of referent biologics prices. On average, the slopes of reference biologics prices decreased annually by $16.5 (82.1%), $17.98 (1658.4%), $271.3 (215.8%), $15.5 (87.3%), $54.7 (123.8%), and $2.8 (106.2%) for adalimumab, bevacizumab, filgrastim, infliximab, pegfilgrastim, and trastuzumab, respectively. Price differences between reference and biosimilar adalimumab and epoetin after biosimilar market entry varied by country income levels. The average price of reference adalimumab was $28.8 (40.9%) and $149.3 (84.7%) higher than biosimilar adalimumab in upper middle-income and high-income countries, respectively. Similarly, the average price of branded epoetin was $21.4 (51.9%), $29.9 (77.4%), and $122.2 (126.5%) higher than biosimilar epoetin in lower-middle, upper-middle, and high-income countries, respectively.

**Fig 3:**
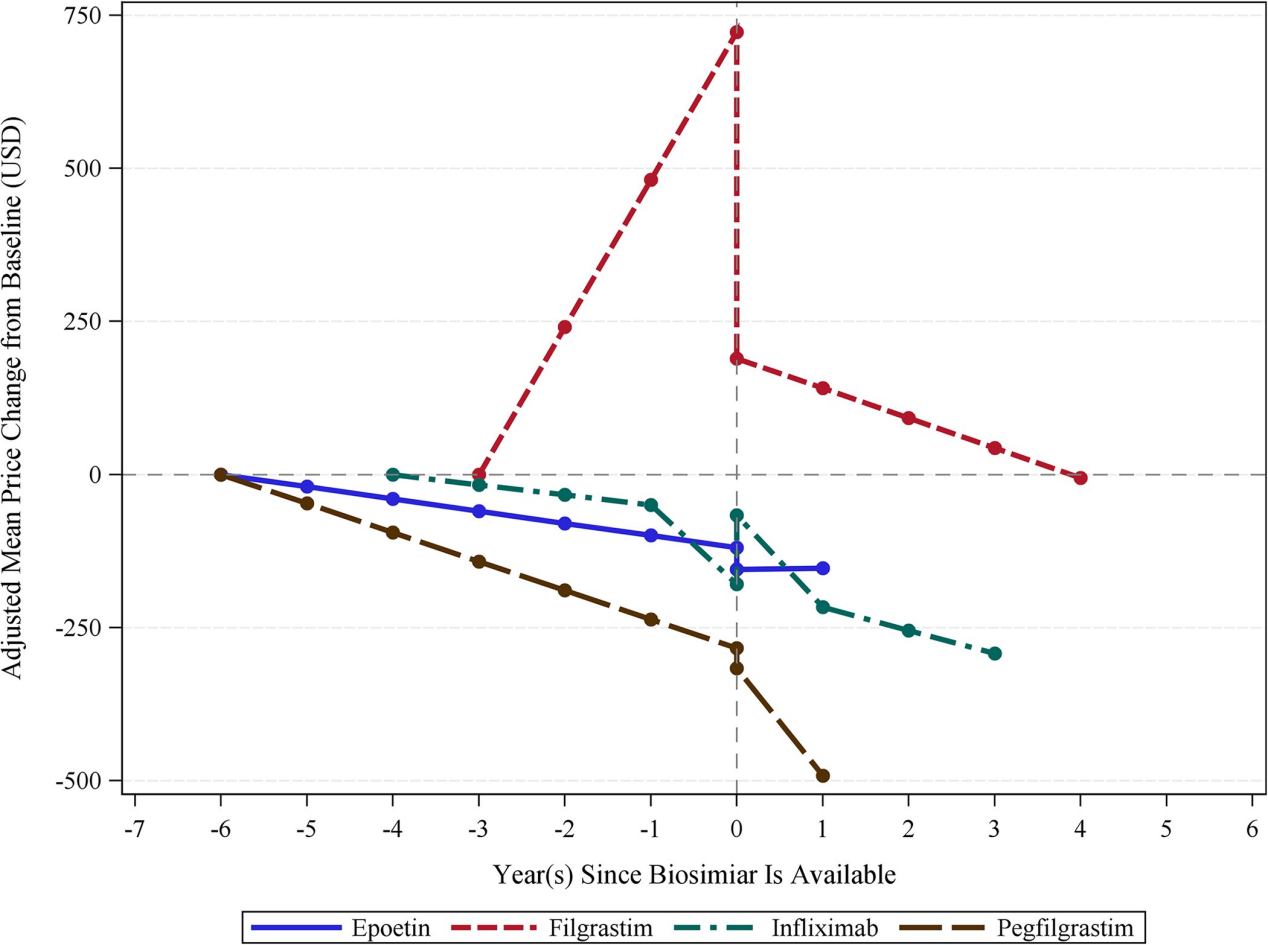
Average biologic medication prices before and after biosimilar introductions in the United States. Source: This is based on internal analysis by Hui-Han Chen, Tatenda Yemeke, and Sachiko Ozawa using data from the following source: IQVIA MIDAS Annual Sales for the period 2012–2019 reflecting estimates of real-world activity. Copyright IQVIA. All rights reserved.

Fig 3 presents an analysis specific to the US, demonstrating the impact of the market entry of biosimilar epoetin, filgrastim, infliximab, and pegfilgrastim in the US. We found that biosimilar entry instantly reduced average prices of epoetin, filgrastim, and pegfilgrastim by $44.9 (18.8%), $1523.2 (50.3%), and $203.1 (4.2%), respectively, and increased the average price of infliximab by $44.5 (5.4%), although these instant changes were not statistically significant. In contrast, the ITS analysis showed that filgrastim, infliximab, and pegfilgrastim had a significant change in the slope of average prices over time. The long-term slope of biologics prices showed annual reductions of $955 (217.6%) for filgrastim, $104.4 (419.5%) for infliximab, and $752.5 (430.6%) for pegfilgrastim over the six years since biosimilars became available.

## Discussion

The study findings demonstrated that the average price of biologics significantly reduced after the introduction of biosimilars across different countries and regions. Specifically, this analysis showed that the entry of biosimilars impacted the biologics price through both instant reductions and changes in long-term price trends. Three of the eight biologics (i.e., bevacizumab, infliximab, and trastuzumab) had a significant instant reduction in price. Biosimilars were more important in reducing the long-term price trends for adalimumab, filgrastim, infliximab, and trastuzumab. Notably, we also observed a similar influence of long-term price reductions in the US, where three of the four biologics showed a significant reduction in the slope of ex-manufacturer prices over time.

Among the eight biologics analyzed in this study, the market entry of biosimilars had the most substantive impact on trastuzumab’s price through an immediate price reduction of $438.4 (27.7%) and a decrease in the post-intervention slope of the price over time by $189.3 (484.2%) per year. The study results demonstrated that the introduction of biosimilars was associated with ex-manufacturer medication prices. We observed a notable increase in biologics manufacturers following the introduction of a biosimilar into the market, which was likely to be associated with reduced average prices for biologics due to increased market competition. This finding is consistent with earlier studies showing that the number of generic manufacturers is a good predictor of the average drug price [39].

Our study results are consistent with previous publications on the impact of biosimilars on drug prices. It is estimated that biosimilar entry reduced the price of biologics by around 40% in some European countries [40, 41]. In the US, Medicare spending on biologics reduced by 26.6% from 2015 to 2019 due to biosimilars’ entry, and competition from biosimilars is projected to save $24 to $150 billion in total biologics expenditures between 2017 and 2026 [29, 42]. Prior studies indicate that biologics’ price reduction due to biosimilars’ entry can be achieved through two different pathways [43, 44]. First, the change in average drug price is driven by increased use of cheaper alternatives, which reduces the utilization of the more expensive brand-name biologics [23, 30]. Due to reduced expenditures on brand-name biologics, substituting reference products with biosimilars reduces the average price of biologics [27]. We have observed an increase in the overall consumption of biologics, including both reference and biosimilar products, across six biologics. Notably, there was a decrease in the demand for reference products one year after the introduction of biosimilars. Meanwhile, the utilization of biosimilars increased over time for seven out of eight biologics in the post-introduction period. These findings support the proposed mechanism that substituting brand-name biologics with more affordable biosimilars would lead to a reduction in the average price of biologics. Second, the manufacturers of reference biologics tend to reduce the price of their products to counteract the weakening of sales due to competition from biosimilars [43]. Our study results also align with this mechanism, where we observed that the average prices of reference biologics tend to reduce immediately after the entry of biosimilars. Similarly, prices of reference biologics kept decreasing over time after the entry of their biosimilars. This suggests that competition from the availability of biosimilars led to the instant and long-term effects of reduced prices of reference biologics.

In addition, we observed that the European Region stood out as one of the most significant markets for biosimilars, with most of the sales data in this analysis originating from European countries. These findings align with a prior study, indicating greater availability of biosimilars in European nations as compared to the US [45]. An important factor contributing to the availability of biosimilars is the landscape of medication patent protection [46, 47]. A prior study suggests that bevacizumab, infliximab, and rituximab have over 90 patents in the US [47]. By leveraging this patent protection, manufacturers of reference biologic products can achieve agreements with biosimilar manufacturers to postpone the market entry of biosimilars. For example, despite the high volume of adalimumab use in the US, several biosimilar adalimumab approved by the FDA have yet to enter the market due to patent disputes [47]. These differences in patent regulations can limit the availability of biosimilars in the US as compared to Europe.

This study has several limitations. First, the IQVIA MIDAS data offer prices paid by wholesalers and distributers, differing from the actual amounts paid by patients and healthcare payers for medications. Biologic manufacturers might offer additional rebates to different distribution channels to enhance their competitiveness in the market, and these rebate amounts may not be reflected in the IQVIA data, which operates at the ex-manufacturer level. Therefore, the findings of this study may not be generalizable to the costs that patients and healthcare payers incur directly. Second, our analysis did not control for personal-level demographic variables as the data did not contain further information such as age and indication. Third, our study did not analyze data from all countries and all biosimilars. However, as we systematically analyzed all data available in the IQVIA MIDAS dataset, the study results still have merit in investigating the impact of the market entry of biosimilars on price reductions across different biologics across a variety of countries. Lastly, the results presented in this study may be subject to other unobserved time-variant confounders. Further comparisons across countries should consider the important variants in the structure of each country’s healthcare system, drug pricing policies and regulations, and the use of biologics in the country’s treatment guidelines.

## Conclusion

The results of this study indicate the importance of biosimilars in containing healthcare expenditures based on the analyses of transaction prices between manufacturers and distribution channels after the market entry of biosimilars in the US and worldwide. Evaluating the instant and long-term changes in biologic prices by making biosimilars available is the first step toward capturing the benefits of increasing biosimilar utilization. Our results demonstrate the effect sizes of the instant and long-term changes in biologic prices due to biosimilar entry across different countries and years. This study highlights the need for global policymakers to appreciate the value of biosimilars in reducing the costs of biologics and improving patients’ access to effective treatments. By demonstrating the significant cost reductions in average biologic prices that biosimilars can trigger after market entry, our study estimates can help inform policies to improve biosimilars’ availability and curb the growing medical spending in the global biologics market.
